# Three Interesting Cases of Hypoglycemia: A Case Series

**DOI:** 10.7759/cureus.100688

**Published:** 2026-01-03

**Authors:** Binod Prusty, Sambit Das, Bijay Das, Dayanidhi Meher, Vishal Agarwal, Arun Choudhury, Sandeep K Sahu, Devadarshini Sahoo

**Affiliations:** 1 Endocrinology, Diabetes and Metabolism, Kalinga Institute of Medical Sciences, Bhubaneswar, IND

**Keywords:** endogenous hyperinsulinemic hypoglycemia, factitious hypoglycemia, hypoglycemia, insulin autoimmune syndrome, pancreatic insulinoma

## Abstract

Hypoglycemia can be due to exogenous hyperinsulinemic hypoglycemia or endogenous hyperinsulinemic hypoglycemia (ENHH). ENHH is a rare entity characterized by inappropriately elevated plasma insulin in the background of low plasma glucose levels. ENHH, being an uncommon disease, is caused by many rare entities, with drug-induced being the most common cause of hypoglycemia. Other causes include insulinoma, insulin autoimmune syndrome, and nesidioblastosis. Here, we report three rare cases of non-diabetic hypoglycemia referred to the endocrinology clinic who had signs and symptoms of hypoglycemia fulfilling Whipple's triad with evidence of hyperinsulinemia despite low plasma glucose. All the appropriate biochemical investigations along with imaging studies were conducted to find out the etiology of hypoglycemia. After taking detailed medical history and clinical examination with biochemical and radiological investigations, patients were diagnosed with insulin autoimmune syndrome, insulinoma, and drug-induced hypoglycemia. Appropriate management was started, and patients were relieved of hypoglycemia. Diseases causing hypoglycemia have similar manifestation, but the diagnosis and management are always a challenging task. Hence, an accurate and early diagnosis is always a deciding factor for further prognosis.

## Introduction

Hypoglycemia is clinically defined as plasma glucose concentrations low enough to cause symptoms or signs including alteration in brain function. Many mechanisms exist in our body that maintain this narrow range of blood sugars. Neuronal and counter-regulatory hormonal mechanisms worked hand in hand to prevent hypoglycemia [1]. In general, for people who are not on glucose-lowering agents, hypoglycemia is an uncommon event [2]. Diagnostic criteria for defining hypoglycemia, popularly known as Whipple's triad, is used to decide whether further evaluation for hypoglycemia is needed or not [3]. Documentation of Whipple's triad is crucial for diagnosing hypoglycemia which includes symptoms, signs, or both consistent with hypoglycemia, a reliably measured low plasma glucose concentration, and the resolution of symptoms or signs after raising the plasma concentration. Hypoglycemia is commonly seen in diabetes but is a rare entity in the absence of diabetes. In a healthy individual, symptoms of hypoglycemia develop when the mean plasma glucose concentration falls below 55 mg/dl [4]. Hypoglycemia in healthy adults can be drug-induced, associated with critical illnesses, and due to a deficiency of counter-regulatory hormones and other rare causes like endogenous hyperinsulinemic hypoglycemia (ENHH).

ENHH is a rare condition marked by low blood sugar levels brought on by an overabundance of endogenous insulin. Palpitations, hunger, diaphoresis, disorientation, and even unconsciousness might be symptoms of hypoglycemic episodes. Symptoms of hypoglycemia manifest as neurogenic (autonomic symptoms) and neuroglycopenic symptoms which can be dreadful leading to seizure and coma. Early diagnosis and treatment of hypoglycemia in healthy individuals leads to a substantial decrease in morbidity and mortality. Here, we present three rare case reports of hypoglycemia. The first case is an uncommon case of insulin autoimmune syndrome (IAS). According to current data, it is more prevalent in Japanese individuals, with only 28 documented cases in India as of 2020 [5]. Also, in the absence of common drug triggers, it is possible that the Indian population is susceptible to novel environmental or idiopathic causes. Similarly, the second case is a case of insulinoma which is an extremely rare cause of hypoglycemia with a reported incidence of 0.7-4 cases per million per year. Lastly, the third case highlights the crucial role of psychiatric and endocrine examination in diagnosing a case of factitious hypoglycemia (FH).

## Case presentation

Case 1

A 55-year-old man was referred to the endocrinology department with chief complaints of recurrent episodes of hypoglycemia. For approximately one year, he has been experiencing episodes of tingling sensations in both upper limbs, tremulousness, blurring of vision, dizziness, and generalized weakness. There were no episodes of excessive perspiration or loss of consciousness. These symptoms disappeared with frequent meals. At the onset of symptoms, he had documented random plasma glucose of less than 3 mmol/l (<55 mg/dl). There was no history suggestive of hepatic failure, renal failure, sepsis, or glucocorticoid deficiency, and no other family members were suffering from diabetes or a similar kind of illness. On examination, his blood pressure was 126/82 mmHg, with a pulse rate of 88 per minute with no pallor or jaundice, and the systemic examination was unremarkable. Following admission, all routine investigations, including complete blood count, kidney function test, liver function test, 8 am serum cortisol, serum growth hormone, and thyroid profile, were sent, and the results are described in Table 1.

**Table 1:** Biochemical and hormonal profile of the cases. SGOT: serum glutamic-oxaloacetic transaminase; SGPT: serum glutamate pyruvate transaminase; ALP: alkaline phosphatase; GGT: gamma-glutamyl transferase; TSH: thyroid-stimulating hormone; HbA1c: glycated hemoglobin; RBS: random blood sugar; GH: growth hormone

Parameters	Case 1	Case 2	Case 3	Reference range
Hemoglobin	12.5	13	11.4	11.5-15.5 g/dl
Total leucocyte count	6700	9000	5400	4000-10000/µl
Total platelet count	175×10^3^	150×10^3^	188×10^3^	150-410×10^3^/µl
Serum urea	22	18.8	18	12-42 mg/dl
Serum creatinine	0.9	1.1	0.56	0.7-1.3 mg/dl
Serum sodium	136	136	138	135-145 mmol/l
Serum potassium	3.8	3.7	4.1	3.5-5.1 mmol/l
Serum calcium	2.22	2.27	2.27	2.15-2.57 mmol/l
Serum phosphorus	1	1.19	0.90	0.8-1.45 mmol/l
Serum albumin	4.1	4.6	4.0	3.9-4.9 g/dl
SGOT	30	30	20	0.0-40 U/l
SGPT	36	28	26	5-40 U/l
ALP	23	54	34	40-129 U/l
Serum bilirubin	0.8	1	1.1	0.2-1.2 mg/dl
GGT	57	16	45	10-60 U/l
Serum TSH	2.8	2.5	3.1	0.35-4.2 µIU/ml
Serum cortisol	515.8	736.557	488.2	184.8-623.4 nmol/l
HbA1c	5.8	3.7	5.9	>6.5%
Fasting blood glucose	4.32	1.722	1.55	3.88-7.77 mmol/l
RBS	1.33	2	1.11	3.88-7.77 mmol/l
Serum C-peptide	2.45	1.874	1.85	0.238-1.21 nmol/l
Serum insulin	240.38	83.24	90.38	1.9-23 µIU/ml
Blood ketone bodies (beta-hydroxybutyrate)	0.0038	0.03	0.0023	0.02-0.26 mmol/l
Serum GH	0.784	0.790	-	0.03-2.47 ng/ml
Serum insulin autoantibody	1120	Negative	1.1	<10 U/ml

The patient was kept on observation and monitored for signs and symptoms of hypoglycemia with capillary blood glucose (CBG) monitoring every two hours. A critical sample was drawn when the patient had signs or symptoms of hypoglycemia with a CBG of less than 3 mmol/l (<55 mg/dl). Subsequently, the patient was treated for hypoglycemia with intravenous dextrose. The critical sample includes random blood glucose, fasting insulin, serum C-peptide, blood ketone bodies, and serum insulin autoantibody. He developed hypoglycemic symptoms four hours after taking food, and the results of CBG are summarized in Table 2. His complete blood count, serum cortisol, renal function, and liver function test were normal. The presence of elevated fasting insulin and increased serum C-peptide with positive serum insulin autoantibody clinched the diagnosis of IAS, also known as Hirata syndrome. The patient was managed with lifestyle modifications like small, frequent, low-carbohydrate meals and also bedtime or late-night meals to avoid hypoglycemia, and pharmacotherapy like glucocorticoids and alpha-glucosidase inhibitors was advised. The patient on subsequent follow-up after three months had no symptoms of hypoglycemia and had clinically improved, and glucocorticoid doses were subsequently tapered.

**Table 2:** CBG monitoring. CBG: capillary blood glucose

Time	Test values of CBG (reference range: 3.88-7.77 mmol/l)
10:00 AM	5.55 (100)
12:00 PM	6.0 (108)
2:00 PM (food taken)	9.33 (168)
4:00 PM	2.0 (36)

Case 2

**Figure 1:**
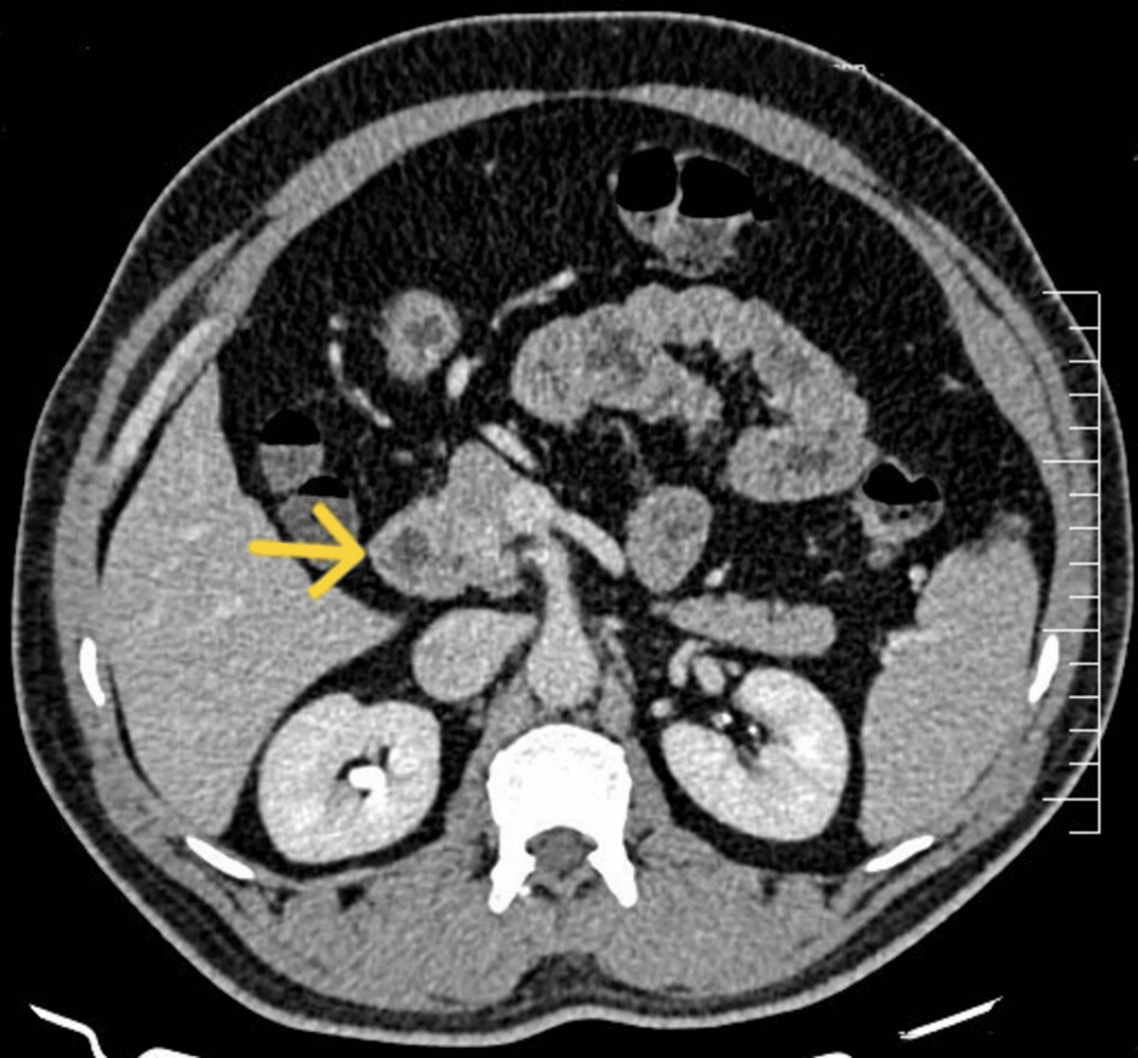
CECT of the abdomen demonstrating an ill-defined, heterogeneously hyperenhancing 21×15 mm lesion in the pancreatic head (arrow), consistent with insulinoma. CECT: contrast-enhanced computed tomography

A 54-year-old man presented to the endocrinology outpatient department (OPD) with recurrent episodes of signs and symptoms of hypoglycemia that have been bothering him for over a year. Symptoms such as palpitations, headache, head reeling, sweating, anxiousness, blurred vision, and blackouts were more common during the early morning while fasting. These symptoms typically improve after consuming calorie-containing fluid/substances. On one occasion, he developed head reeling and subsequently lost consciousness and was immediately taken to a nearby local hospital, where he was treated with 25% dextrose, as his CBG monitoring at that point in time was 1.33 mmol/l. He was admitted to the endocrinology department for the evaluation of hypoglycemia. Post-hospitalization, a general physical examination was done, which was essentially normal. Routine investigations were ordered to rule out any systemic cause of hypoglycemia, which also turned out to be normal. The patient was monitored closely for the development of signs and symptoms of hypoglycemia. Once the patient developed signs and symptoms of hypoglycemia, a CBG was done, which turned out to be 2.55 mmol/l. Critical samples were sent, which revealed a random blood sugar (RBS) of 1.99 mmol/l. All the biochemical investigations are tabulated in Table 1. The patient was subsequently subjected to contrast-enhanced computed tomography (CECT) of the abdomen. CECT revealed an ill-defined lesion in the pancreatic head consistent with an insulinoma which has a sensitivity of 54% and specificity of 75% (Figure 1). With these biochemical parameters showing elevated fasting insulin and serum C-peptide with suppressed serum ketone bodies and radiological imaging depicting a pancreatic mass, a provisional diagnosis of endogenous hyperinsulinemia (insulinoma) was made, and the patient was referred to the surgical oncology department for surgical resection of the pancreatic lesion. Till then, he was managed conservatively with small, frequent, low-carbohydrate meals.

Case 3

**Figure 2:**
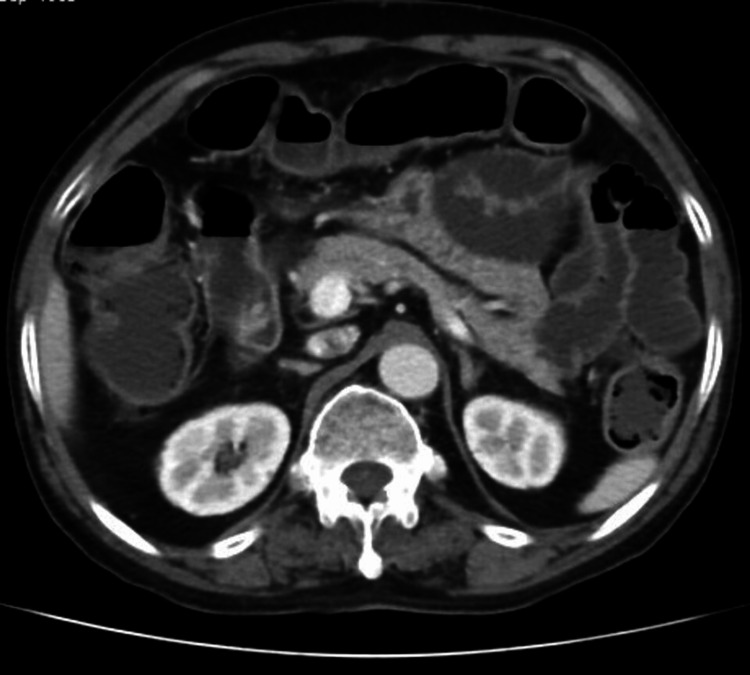
CECT of the abdomen showing normal pancreas morphology. CECT: contrast-enhanced computed tomography

**Figure 3:**
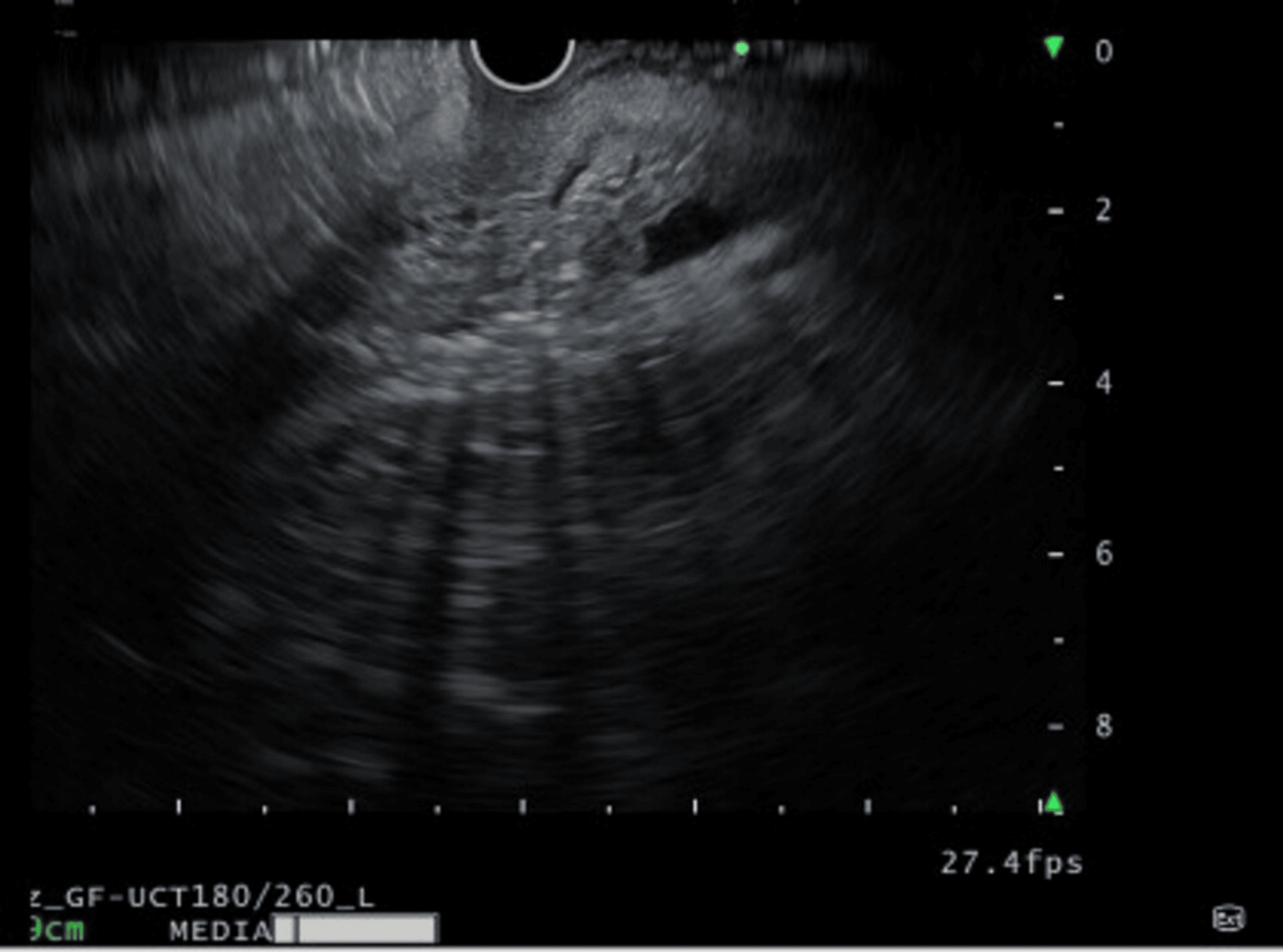
EUS showing normal pancreas with homogeneous echotexture without any focal masses. EUS: endoscopic ultrasound

A 57-year-old married woman presented to the emergency department with multiple episodes of syncopal attacks associated with blurring of vision, dryness of mouth, paresthesia in bilateral upper limbs, diaphoresis, and palpitation. She had these symptoms in repeated episodes for the last month and was managed with intravenous dextrose-containing fluids at a local hospital, following which her symptoms got relieved and she was discharged. But since her frequency of symptoms gradually increased, she was referred to a tertiary center for proper evaluation. After admission to the emergency department, on examination, she had tachycardia, with a pulse rate of 110 per minute and a blood pressure of 124/82 mmHg, and was sweating profusely, and the rest of her systemic examination was unremarkable. A random blood glucose was sent, which showed 1.88 mmol/l. The patient was started on intravenous 25% dextrose with CBG monitoring done every two hours, but still the symptoms persisted. Hence, she was simultaneously started with 10% dextrose intravenous fluid at 100 ml/hr, keeping the target CBG at 5.55-9.99 mmol/l. After starting conservative management, she maintained blood glucose within the target range, was withdrawn from all intravenous fluids, and was advised to take them orally, but after stopping treatment for 24 hours, she again developed similar symptoms with a random blood glucose of 2.55 mmol/l. This raised an alarming situation, and the patient was referred to the endocrinology department for the evaluation of hypoglycemia. All routine investigations consisting of a complete blood count, kidney function test, liver function test, 8 am serum cortisol, thyroid profile, and serum insulin autoantibody were sent, and the results are summarized in Table 1. CBG monitoring was done every two hours, and a critical sample was sent when the patient developed signs and symptoms of hypoglycemia. Because of the persistence of hypoglycemia in the background of hyperinsulinemia, she was suspected to have hyperinsulinemic hypoglycemia with unknown etiology. CECT of the whole abdomen (Figure 2) and endoscopic ultrasound (EUS) (Figure 3) showed no pancreatic or extra-pancreatic lesions. The patient was diagnosed as a case of hyperinsulinemic hypoglycemia in view of elevated fasting serum insulin, serum C-peptide with low serum ketone bodies, and absent serum insulin autoantibody in the background of normal imaging. With the history of her husband on glibenclamide for type 2 diabetes mellitus, a suspicion of FH was made. The patient denied intake of any hypoglycemic agents. However, after repetitive sessions with a psychiatrist, she agreed to the intake of glibenclamide which her husband was taking, and this behavior was driven by her husband's disability because of arthritis which subsequently made her depressed. The patient was kept under observation, keeping her blood sugars in the target range, and further psychiatric consultation was taken for managing her depressive personality.

## Discussion

Hypoglycemia is usually a phenomenon which is often seen as an adverse effect of drugs taken to treat diabetes. Sulfonylureas and insulin are the widely used medications that have been known to cause hypoglycemia. Many mechanisms exist in our body that maintain this narrow range of plasma glucose. Before evaluating any case of hypoglycemia (in patients who are not on glucose-lowering agents), it is prudent to rule out systemic causes like sepsis, hepatic failure, renal failure, etc. which are a few of the common causes that can lead to hypoglycemia at the outset before subjecting the patient to unnecessary investigations.

Hence, as per the Endocrine Society Clinical Practice guidelines, patients developing hypoglycemia are categorized based on their clinical status, i.e., patients who are seemingly well and ill-appearing patients [4]. In patients who are seemingly well, it is further subclassified into the following: (a) endogenous hyperinsulinemia (insulinoma, post-bariatric hypoglycemia, noninsulinoma pancreatogenous hypoglycemia), (b) insulin autoimmune hypoglycemia (antibody to insulin and antibody to insulin receptor), and (c) accidental, factitious, or malicious hypoglycemia [3].

ENHH is a very rare disease characterized by hypoglycemia in association with endogenous hyperinsulinemia. The diagnosis of ENHH is based on random plasma glucose <55 mg/dl (<3 mmol/l), plasma insulin ≥3 µU/ml, and plasma C-peptide >0.6 ng/ml [6,7]. Now, let us discuss in detail about our individual cases.

IAS is characterized by hyperinsulinemic hypoglycemia in the presence of high levels of circulating insulin autoantibodies in the plasma. IAS is also known as Hirata's disease coined after Yukimasa Hirata and colleagues in the year 1970. The estimated prevalence of IAS is 0.017 cases per 100,000 in the general population as per a study conducted in 2019 among the Japanese population. IAS is a syndrome seen in patients who are not previously exposed to exogenous insulin. It is usually diagnosed in the fourth decade of life in women and in the seventh decade of life in men [8].

The exact etiopathogenesis of IAS is unknown, but the widely accepted theory is the association of genetic predisposition with environmental triggers which leads to the development of IAS. IAS is strongly associated with class II human leukocyte antigen (HLA), i.e., HLA-DRB1*0406 [9], and environmental triggers more commonly drugs, viral infection, and hematological disorders. Methimazole and alpha-lipoic acid are the most common drugs associated with IAS. Many viral infections like mumps, rubella, measles, and the hepatitis C virus are reported to trigger the development of IAS.

Insulin autoantibody has the property of high binding capacity with low affinity which increases the half-life of insulin from five minutes to a few hours that induces the development of IAS. Patients affected with IAS present with high concentrations of plasma insulin as seen in our case 1 (Table 1).

After confirming hyperinsulinemic hypoglycemia, it's the level of C-peptide that differentiates between exogenous and endogenous forms of hyperinsulinemia. Higher or inappropriately normal C-peptide is found in endogenous hyperinsulinemia, whereas lower C-peptide is found in exogenous insulin-induced hypoglycemia.

After the confirmation of endogenous hyperinsulinemia, the differential diagnosis can be insulinoma or IAS, but the presence of serum insulin autoantibody as seen in case 1 confirms the diagnosis of IAS. The treatment of IAS is based on conservative management with the removal of drugs that have triggered the development of IAS. Besides pharmacotherapy, dietary modifications play a primary role in avoiding hypoglycemia. IAS has higher rates of spontaneous remission. Modifying one's diet and lifestyle to lessen the stimulation for insulin secretion is part of managing IAS. It has been discovered that eating small, frequent meals with a low glycemic index and uncooked corn starch reduces the incidence of hypoglycemia episodes.

Pharmacotherapy includes treatment with alpha-glucosidase inhibitors for the prevention of postprandial hypoglycemia. Owing to the autoimmune nature of IAS, higher doses of oral glucocorticoids are associated with favorable outcomes. Other immunosuppressive agents like azathioprine and rituximab can also be used as a steroid-sparing regimen for the management of refractory IAS. Other strategies include a decrease in insulin release by the use of somatostatin analogues or diazoxide. Plasmapheresis can also be used as a treatment modality in very severe cases with the aim to reduce insulin autoantibody titers, thus preventing hypoglycemia. Plasma cell-directed therapy has a promising role in treating severe IAS that is resistant to immunosuppressive or lifestyle therapies, according to recent studies on IAS treatment approaches.

Insulinomas are an extremely rare cause of ENHH with a reported incidence of 0.7-4 cases per million per year [10]. It is reportedly seen in the fifth decade of life in men and the sixth decade of life in women. However, the incidence is slightly more common in women [11]. Most of the insulinomas (>99%) are localized to the pancreas [12]. Extra-pancreatic and metastatic insulinomas have been described in the lungs, jejunum, ileum, gastric antrum, spleen, etc. [13]. Currently, insulinomas are classified into indolent and aggressive. Those insulinomas which metastasize are referred to as aggressive. The majority of insulinomas reported belong to the indolent category which has a five-year survival rate of 94-100% as compared to the aggressive variant with a five-year survival rate of 24-67% [14]. Around 5-10% of patients with insulinoma are associated with the multiple endocrine neoplasia type 1 (MEN-1). Insulinomas can present as solitary lesions or can also present as multiple lesions [15]. There is no consensus on whether to screen for MEN-1 in patients with newly detected insulinoma. NF-1 and tuberous sclerosis are the other genetic syndromes which have been seen to be associated with insulinoma [16,17].

The pathophysiology of insulinoma is poorly understood. Chromatin remodeling is believed to be the underlying pathogenesis for insulinoma. The mutation in the Yin Yang 1 gene is the predominant genetic abnormality seen in around 30% of insulinomas [18]. Loss of heterogeneity of the MEN-1 region at chromosome 11q13.1 is also seen in 30% of insulinomas [19]. Aberrant methylation patterns of the insulin promoter are the other known genetic mechanism that leads to the development of insulinoma [20]. The clinical presentation of insulinoma is similar as described above for Hirata's disease. However, unlike Hirata's disease, hypoglycemic episodes are more commonly seen in the fasting state. However, postprandial hypoglycemia has also been reported. Like in Hirata's disease, the diagnosis is established through biochemical investigations by testing the critical sample which is drawn when the patient develops signs and symptoms of hypoglycemia with the CBG value being less than 55 mg/dl.

For the localization of the insulinoma, CECT is the modality of choice; however, EUS or magnetic resonance imaging (MRI) can be used if CECT is nonconclusive. Recently, the use of glucagon-like peptide-1 receptor (GLP-1R) positron emission tomography/computed tomography (PET/CT) or PET/MRI has gained popularity for the localization of indolent, localized, and occult insulinomas [21]. However, if the insulinoma is aggressive, which usually expresses somatostatin receptor 2 (SSTR2), PET/CT or PET/MRI using 68 Ga-DOTA-labeled somatostatin receptor ligands (SRL) can be used. Earlier, somatostatin receptor scintigraphy and single-photon emission computed tomography (SPECT) using ^111^In-pentetreotide were used [22,23]. In high-volume centers, selective pancreatic angiography and selective intra-arterial injection of calcium with hepatic vein insulin were routinely used in the past. However, due to the recent advances in the imaging modalities, this procedure is seldom used [24]. 

The primary modality of the treatment is surgery. Surgical resection can be carried out if the tumor is localized and is solitary. Enucleation can be carried out provided the insulinoma is small in size (<2 cm) and the lesion is located >2-3 mm from the main pancreatic duct or the common bile duct [25]. Patients who have not had a significant improvement from invasive procedures, are nonsurgical candidates, or are awaiting surgery should be evaluated for medical management. The medical treatment in the form of diazoxide which is a non-diuretic benzothiadiazine vasodilator that decreases insulin secretion can be tried [26]. Other modalities include SRL such as lanreotide and octreotide [27], and glucocorticoids and drugs like phenytoin, diltiazem, and verapamil which have beta-adrenergic receptor blocking activities can also be used in selected cases [28]. Peptide receptor radionuclide therapy with radiolabeled SRL is currently an established second line of therapy in aggressive insulinoma which are well differentiated (tumor ki67 index ≤20%) [29]. Mammalian target of rapamycin such as everolimus has also been used in the treatment of metastatic neuroendocrine tumors (NETs) owing to its anti-proliferative properties [30]. In our case, the second patient was diagnosed as ENHH with negative serum insulin autoantibody with a provisional diagnosis of insulinoma, which was later confirmed on CECT of the abdomen.

FH is the intentional use of oral hypoglycemic medications or insulin preparations to lower blood glucose levels [31]. When diagnosing non-diabetic hypoglycemia (NDH), the possibility of FH must always be taken into account [4]. Among the several causes of NDH, FH is present in about 10.8% of cases [31]. People who have access to diabetes drugs and/or medical education are more likely to have this illness [31-33]. FH can have a major impact on the clinical picture of a mental health illness and the determination of an accurate diagnosis in patients with such issues [31,34]. FH is one of the most challenging disorders to diagnose in a patient with hypoglycemia [35-37].

A few distinct biochemical indicators of insulin-mediated hypoglycemia are necessary for the diagnosis of insulin and insulin secretagogue-induced hypoglycemia [4]. Low levels of C-peptide and pro-insulin indicate that endogenous insulin secretion is inhibited in the context of exogenous insulin administration and normal pancreatic function. Hepatic glycogen reserves are maintained, while ketone synthesis is inhibited. Insulin secretagogue-induced hypoglycemia, on the other hand, is characterized by endogenous insulin synthesis that is continued, as seen by high or improperly non-suppressed levels of pro-insulin, C-peptide, and insulin. Similarly, the liver's glycogen stores are maintained, while ketone synthesis is inhibited. Serum or urine testing for oral hypoglycemic drugs is crucial for an appropriate diagnosis because the biochemical profile of insulin secretagogue-induced hypoglycemia is the same as that of insulinoma or noninsulinoma pancreatogenous hypoglycemic syndrome, as seen in our third case, where the serum insulin, serum C-peptide, and serum ketone bodies were similar to other causes of ENHH, but in the absence of serum insulin autoantibody, with no abnormal radiological imaging, and with a positive history of intake of oral hypoglycemic drugs, a diagnosis of FH was made.

In summary, in the presence of elevated insulin, C-peptide with a normal or low (<1) serum insulin-to-C-peptide ratio suggests insulinoma- or sulfonylurea-induced hypoglycemia, and a high insulin-to-C-peptide ratio favors IAS or hypoglycemia due to exogenous insulin.

In the end, treating hypoglycemia caused by insulin secretagogue and exogenous insulin over the long term mostly entails addressing the root cause by discontinuing the offending medicine. A multidisciplinary approach involving psychiatry and psychology is advised, nevertheless, because FH implies an underlying mental health condition [38].

## Conclusions

Hyperinsulinemic hypoglycemia remains a clinical challenge to be diagnosed. Hypoglycemia causes functional brain failure which gets resolved after the elevation of blood glucose to normal levels. Profound, prolonged hypoglycemia can cause brain death. In addition, it is well known that severe hypoglycemia increases cardiovascular risk and worsens cognitive impairment. An appropriate approach and accurate diagnosis help in avoiding unnecessary invasive procedures and help in preventing complications due to hypoglycemia. As in our case series, the background of hypoglycemia due to hyperinsulinemia with positive insulin autoantibody, pancreatic mass on CT of the abdomen, and history of depression with surreptitious intake of oral hypoglycemic medications are the pointers for the diagnosis of IAS, insulinoma, and FH, respectively.
